# Interplay of singlet and triplet excited states in photoredox catalysis

**DOI:** 10.1039/d6sc04715g

**Published:** 2026-10-21

**Authors:** Caterina Bellatreccia, Federica Fina, Paola Ceroni

**Affiliations:** a Dipartimento di Chimica “Giacomo Ciamician”, Alma Mater Studiorum – Università di Bologna via Pietro Gobetti 85 40129 Bologna Italy paola.ceroni@unibo.it

## Abstract

Photocatalysis has emerged as a rapidly expanding field, offering promising strategies for driving challenging and thermodynamically uphill transformations through the use of light. By harnessing the unique reactivity of electronically excited states, photocatalysis requires the coupling of the rigorous mechanistic approach of photophysics with the synthetic insight of organic chemistry. This perspective provides a conceptual framework for understanding fundamental aspects of photoredox catalysis, with a focus on the competitive roles of singlet (S_1_) and triplet (T_1_) excited states of the photocatalyst in determining overall catalytic efficiency. We analyse how key parameters, including photophysical properties of the photocatalyst (*e.g.*, intersystem crossing efficiency and lifetimes of excited states) and reactant concentration modulate the balance between singlet and triplet-mediated pathways under catalytic conditions. Particular attention is given to how dynamic changes in reaction mixtures and quenching processes can shift the relative contribution of these excited states over time, thereby influencing overall performance. Selected literature examples are used to illustrate how these factors translate into observable differences in reactivity, selectivity, and efficiency across representative photoredox transformations. We thus aim to provide mechanistic insights and kinetic modelling that bridge photophysical understanding and synthetic application, ultimately guiding the rational design of more efficient photoredox systems and optimized reaction conditions.

## Abstract 2

The photoredox reaction pathway is shaped by the competition between singlet (S_1_) and triplet (T_1_) excited states, under the influence of photophysics, reaction conditions, quenching and cage escape.

## Graphical abstract

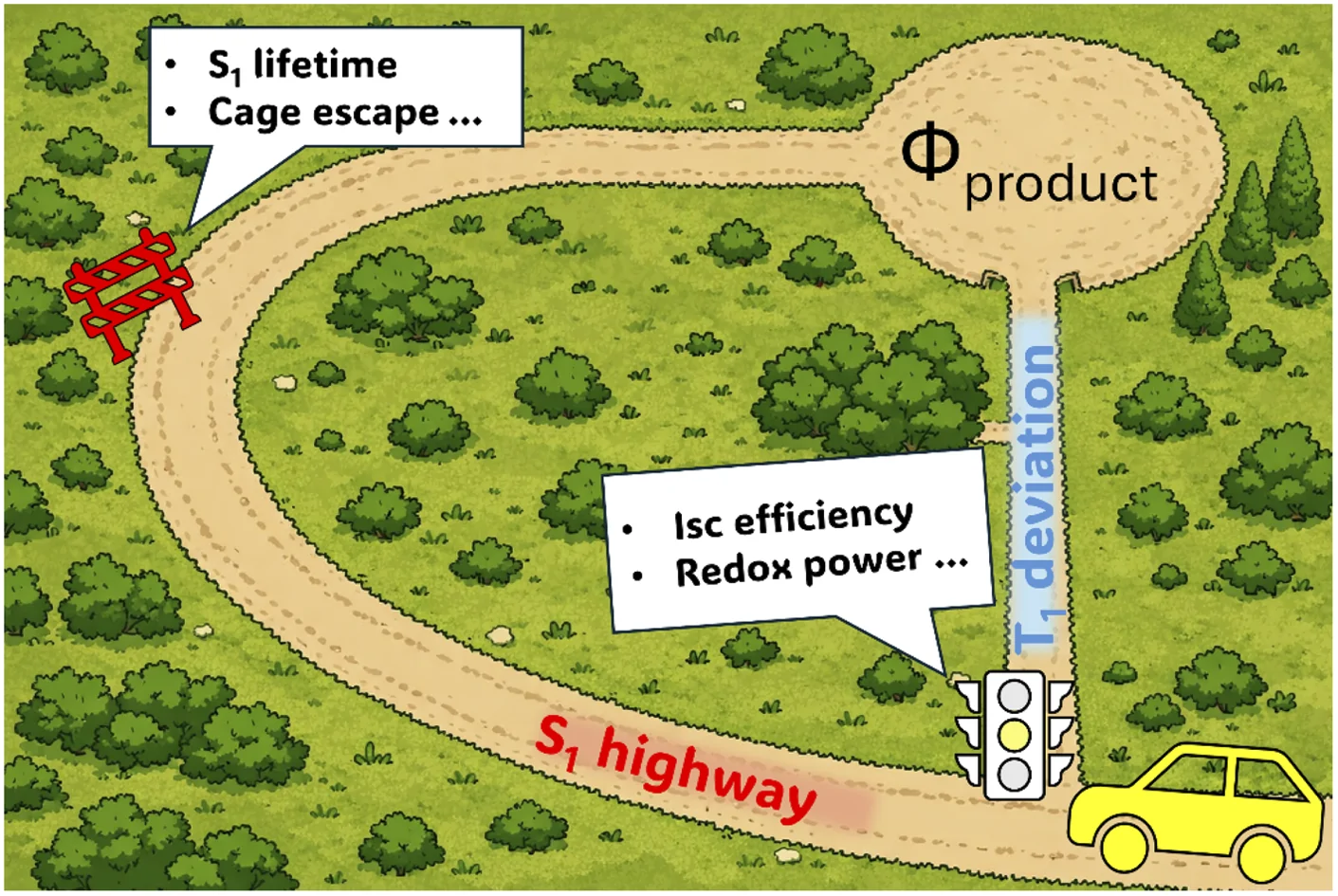

## 1. Introduction

Photoredox catalysis refers to a process in which light is used to promote an electron transfer that is generally thermodynamically uphill in the dark.^1–5^ Light is absorbed by a molecule, usually referred to as photosensitiser or photocatalyst (PC). Importantly, the excited state initially populated upon light absorption is not necessarily the reactive state, as rapid internal conversion and/or intersystem crossing may lead to a lower-energy excited state (typically S_1_ or T_1_) from which reactivity occurs. The photocatalyst is not consumed during the reaction because a subsequent thermal (dark) redox reaction regenerates the molecule in its initial redox state, as illustrated in the case of reductive quenching of *PC by eqn (1)–(3):^6–8^

1 PC + *hv* → *PC

2 *PC + Q → PC˙^−^ + Q˙^+^

3 PC˙^−^ + A → PC + A˙^−^

4 PC˙^−^ + Q˙^+^ → PC + Q

where Q is a quencher, often a sacrificial reagent that is consumed during the reaction, and A is a substrate molecule of the investigated reaction. The reduced species A˙^−^ then evolves to the final product by a series of dark reactions. It is worth noting that productive reactivity (process (3)) competes with back electron transfer (process (4)), which regenerates the ground-state species and might significantly reduce the overall quantum yield, *i.e.*, the ratio between the number of product molecules formed and the number of photons absorbed.^9–12^ Photoredox catalysis has two main fields of application: (i) synthesis of value-added chemicals and (ii) production of solar fuels, also referred to as artificial photosynthesis. The aim of these two processes differs significantly. In the former, the objective is typically the selective formation of a specific product, regardless of the free energy change of the overall chemical reaction. Sacrificial reagents and even high-energy (*e.g.*, UV) light may be employed, if beneficial. Moreover, the overall photocatalytic quantum yield is often not the primary metric and is frequently not reported, with emphasis instead placed on chemical yield. In artificial photosynthesis, the aim is the efficient conversion of sunlight into stored chemical energy, which means that the overall chemical reaction must be endergonic and no sacrificial reagent should be employed in the ideal case. In this case, the overall photocatalytic quantum yield needs to be maximized since the aim of the process is the conversion of sunlight into chemical energy.

Despite their distinct objectives, the two processes are governed by the same fundamental concepts and design principles. In both cases, a mechanistic understanding of the underlying processes can enable the rational design of high-yielding and low-waste reactions.^13^ It is worth noting that the mechanistic understanding of photoredox processes remains elusive,^14–16^ and at times controversial,^17–25^ due to their intrinsic complexity. This complexity arises, in part, from the presence of multiple reactive excited states and from differences in experimental conditions between mechanistic investigations and practical photoreaction setups (for example, lower concentrations of the photocatalyst are employed in the mechanistic study due to intrinsic limitations of photochemical techniques). Moreover, direct comparison of results across different laboratories is often challenging due to the wide variety of experimental indicators employed.^26^

In this review we focus on the differences in the photochemical properties and reactivity of excited states of *PC according to their spin multiplicity. In particular, we will analyze how singlet (S_1_) and triplet (T_1_) excited states contribute differently to electron-transfer pathways and how their relative populations influence reaction quantum yield (Fig. 1). It should be noted that photocatalysts based on transition-metal complexes may exhibit excited states with spin multiplicities other than singlets and triplets. For example, low-spin Fe(iii) complexes can possess doublet ground and excited states, whereas Cr(iii) complexes feature a quartet ground state and may access excited states of different multiplicities. Nevertheless, we focus exclusively on photocatalysts with closed-shell configuration, whose lowest-lying excited states display singlet and triplet multiplicity, as these represent the vast majority of systems commonly employed in photocatalysis. Among these, we consider three classes of photocatalysts: (i) metal complexes showing very high efficiency of population of the T_1_ excited state and thus exclusive photoreactivity from this state; (ii) organic dyes with very low efficiency of T_1_ population, *e.g.* highly fluorescent molecules, in which S_1_ is the only reactive species; (iii) organic dyes in which population of the T_1_ state is efficient and both S_1_ and T_1_ are photoreactive states. Additionally, we will discuss how variations in quencher concentration affect both the kinetics of excited-state deactivation and the overall quantum yield. A recently reported kinetic model^27^ for optimizing reaction conditions will be applied to literature examples for which the system-specific rate constants are known, rather than being used to predict these rate constants from molecular structure as previously done elsewhere.^28^

**Fig. 1:**
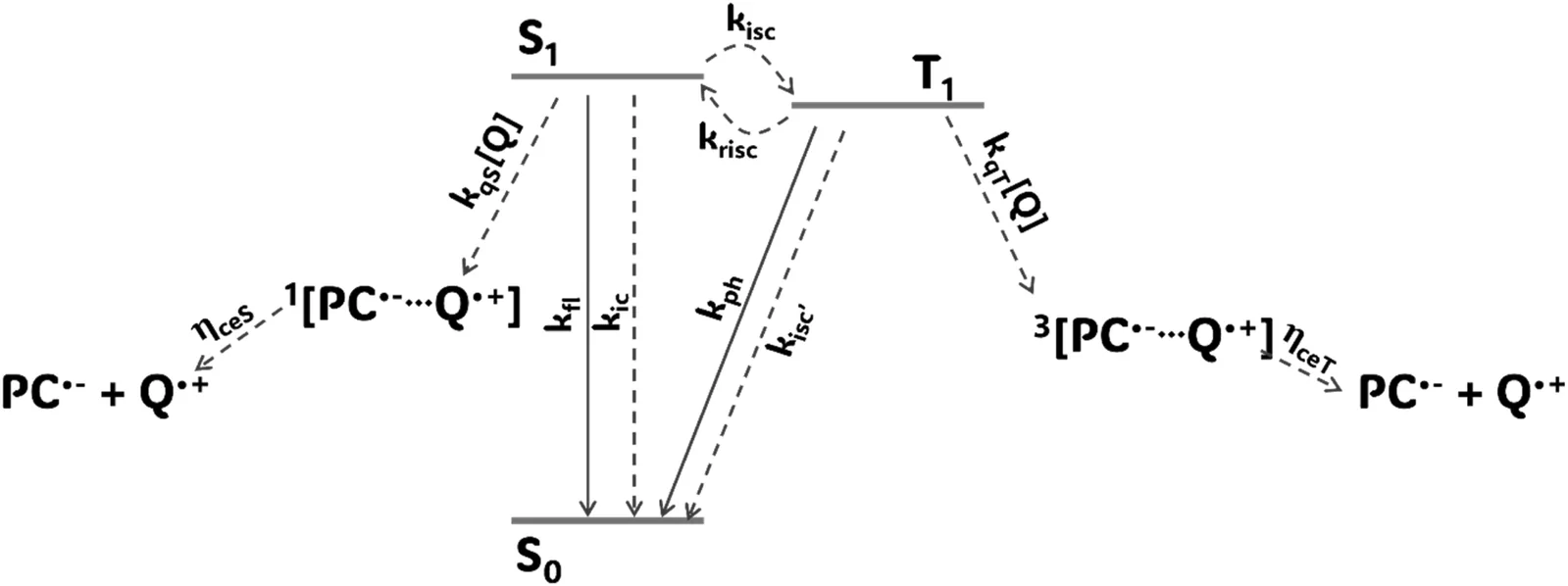
Schematic Jablonski diagram showing the deactivation processes of S_1_ and T_1_ of a photocatalyst (PC) displaying thermally activated delayed fluorescence (TADF). The rate constants of fluorescence (*k*_fl_), phosphorescence (*k*_ph_), internal conversion (*k*_ic_), inter system crossing (*k*_isc_ and *k*_isc′_) and reverse intersystem crossing (*k*_risc_) are indicated, as well as the rate constants of reductive quenching in the presence of quencher Q (*k*_qS_ and *k*_qT_) together with the efficiency of cage escape (*η*_ceS_ and *η*_ceS_). For more details, see text.

## 2. Excited state properties governing photoredox reactivity

The photoreactive excited states of photocatalyst with closed-shell electronic configuration are usually the lowest in energy singlet (S_1_) and triplet (T_1_) excited states. The reason is that the lifetime of higher-energy singlet and triplet excited states is so short (ranging from ps to fs) that the efficiency of reaction (2) would be extremely low for a diffusion-controlled process even at high concentration of the quencher. Indeed, the efficiency of quenching (*η*_q_), *i.e.* number of excited states undergoing a quenching event over the total number of excited states is given by:5
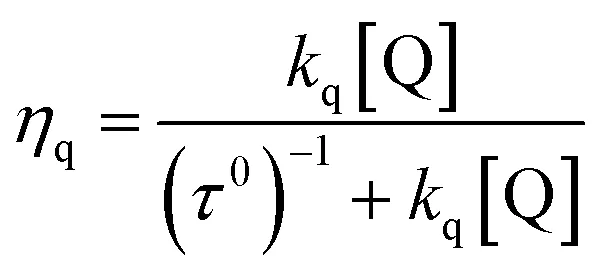
where *k*_q_ is the quenching constant, [Q] is the molar concentration of the quencher and *τ*^0^ is the lifetime of *PC in the absence of Q. For example, if *k*_q_ = 1 × 10^9^ M^−1^ s^−1^, [Q] = 0.1 M and *τ*^0^ = 1 ps, the efficiency of quenching is 0.01%, *i.e.* only 1 in 10 000 *PC molecules is quenched.

Exception to the general assumption that only S_1_ and T_1_ are reactive can be rarely encountered when photocatalysts displaying anti-Kasha behaviour^6^ are employed (this refers to systems where higher-lying excited states participate directly in reactivity) and the quenching is not bimolecular and a complex is formed between PC and Q in the ground state.^29^

Reaction (2) takes advantage of the fact that a molecule in an electronically excited state is a better oxidant and a better reductant than its ground state, which is related to the higher energy of the excited state compared to the ground state. It is worth noting that the main differences between S_1_ and T_1_ are: (i) lifetime, (ii) energy and redox properties, and (iii) cage escape dynamics. The lifetime of S_1_ is much shorter than that of T_1_, usually by 3 orders of magnitude, and this is related to the fact that radiative and non-radiative decay of S_1_ back to the ground state (S_0_) are spin-allowed, while the radiative and non-radiative decay of T_1_ back to S_0_ is spin-forbidden and, thus, slowed down. The energy of S_1_ is larger than that of T_1_ according to the exchange interaction. The magnitude of the energy gap between S_1_ and T_1_ is strictly related to the orbital nature of the transition: it is very large for π, π* transitions like anthracene (1.45 eV) and much smaller for n, π* transition like benzophenone (0.3 eV) or charge-transfer (CT) transition like [Ru(bpy)_3_]^2+^ (0.3 eV).^30^ Having a small energy gap between S_1_ and T_1_ is important to maximize the efficiency of intersystem crossing (isc) between S_1_ and T_1_, which is also promoted by strong spin–orbit coupling (*e.g.*, in heavy-metal complexes). These factors are key in determining whether triplet states are efficiently populated and therefore available for photochemical reactivity. Additionally, a small S_1_–T_1_ gap is essential for observing thermally activated delayed fluorescence (TADF), where S_1_ is repopulated by T_1_*via* thermally activated reverse inter system crossing (risc).^31,32^

The S_1_–T_1_ energy difference is then directly related to their redox properties because the redox properties of an excited state can be evaluated by the following equations:^6^

6 *E*^0^(PC˙^+^/*PC) = *E*^0^(PC˙^+^/PC) − *E*_00_(*PC/PC)/*F*

7 *E*^0^(*PC/PC˙^−^) = *E*^0^(PC/PC˙^−^) + *E*_00_(*PC/PC)/*F*

where *E*_00_(*PC/PC) is the zero–zero spectroscopic energy of the excited state and *F* is the Faraday constant. It is worth noting that *E*_00_(*PC/PC) is related to the reactive excited state that does not necessarily coincide with the excited state populated by light absorption. In other words, when T_1_ is the catalytically active species, its redox properties should be taken into account, using the correct *E*_00_(*PC/PC) value, regardless of the fact that the excitation energy is higher. Furthermore, while these expressions provide a thermodynamic description of excited-state redox potentials, the rates of photoinduced electron transfer are also governed by kinetic factors, including reorganization energy and electronic coupling, as described by Marcus theory.^6^ Being S_1_ higher in energy, it is commonly expected to have higher quenching constants for the electron transfer from S_1_ than from T_1_ due to the higher driving force of the process. In addition to that, when the S_1_–T_1_ energy gap is low – as in photocatalysts diplaying TADF – quenching constants can still significantly differ due to electronic factors, as recently proven for carbazole-based photocatalysts.^33^

The last difference between S_1_ and T_1_ that we highlighted is cage escape dynamics, which can be better explained by considering the intermediate processes occurring in reaction (2), as follows:

8 *PC + Q → (*PC⋯Q)

9 (*PC⋯Q) → (PC˙^−^⋯Q˙^+^)

10 (PC˙^−^⋯Q˙^+^) → PC˙^−^ + Q˙^+^

11 (PC˙^−^⋯Q˙^+^) → PC + Q

where (*PC⋯Q) is the encounter complex and the electron transfer produces the radical pair (PC˙^−^⋯Q˙^+^) within the so-called “solvent cage”; this radical pair then evolves to produce the free radical anion of PC and radical cation of Q. Cage escape^9^ is related to this process (10) that is in competition with the undesired reaction (11), *i.e.* geminate recombination within the solvent cage. The latter produces no net reaction, but decreases the concentration of excited state, slowing down the entire process and it is different from reaction (4), named as back electron transfer, which occurs among free radicals and not within the solvent cage. Cage escape eqn (10) is expected to be more efficient for radical pair generated by the quenching of T_1_ rather than S_1_ states. The reason is that spin multiplicity is retained in the formation of the radical pair, so that a singlet radical pair is formed upon quenching of S_1_ and a triplet radical pair is formed upon quenching of T_1_. Cage escape is in competition with geminate recombination that is favored when singlet radical pair is formed, being a spin-allowed process. By contrast, geminate recombination of a triplet radical pair requires inter system crossing, promoted by spin–orbit or hyperfine coupling.^34^ For simplicity, inter system crossing between singlet and triplet radical pairs is not shown in Fig. 1. It is worth noting that the presence of a magnetic field might affect the quantum yield of production of free radicals, as well as the overall photoreaction quantum yield, as reported for metal complex^35^ and organic photocatalysts.^36–38^

Generally, T_1_ reactivity is preferable over S_1_ reactivity, because of its longer lifetime and its higher cage escape. Having higher cage escape efficiency allows a higher photoreaction quantum yield: a higher fraction of the radical pairs formed will actually evolve into the free radical anion of PC, which subsequently promotes the following reaction steps.^39,40^ On the other hand, as stated before, *E*_00_(*PC/PC) of T_1_ is lower than *E*_00_(*PC/PC) of S_1_, which translates in T_1_ having worse redox properties to be exploited in a photoreaction. In addition to that, in common photocatalysts T_1_ cannot be directly excited, and therefore its utilization in the photocatalytic step requires systems having highly efficient intersystem crossing.

For these reasons, gaining a deep understanding of the whole photochemical cycle and evaluating the conditions under which S_1_ or T_1_ reactivity predominates are crucial for determining the optimal tradeoff among these different factors.

## 3. Metal and organic photocatalysts: photophysical properties

The first class of photocatalysts employed in photoredox catalysis for production of either value-added chemicals or solar fuels was based on metal complexes. The prototypical [Ru(bpy)_3_]^2+^ has been employed both for water splitting processes and for organocatalytic reactions.

At the end of the 1970s, Grätzel *et al.*^41^ and Keller *et al.*^42^ demonstrated hydrogen evolution by light-irradiation of a solution containing the photocatalyst [Ru(bpy)_3_]^2+^, methyl viologen as electron relay, platinum nanoparticles as catalyst and a sacrificial electron donor (aliphatic amine or ethylenediaminetetraacetic acid disodium salt).

Around the same time, Kellogg *et al.*^43^ published the first papers in the field of organic photoredox catalysis:^44^ they reported that the reduction of sulfonium ions by dihydropyridines is enhanced upon light irradiation in the presence of catalytic amounts of [Ru(bpy)_3_]^2+^, tetraphenylporphyrin, or eosin, with the former providing the largest rate enhancement. The field remained relatively inactive until the seminal publication of MacMillan *et al.*^45^ in 2008, in which photoredox catalysis and organocatalysis were merged for the enantioselective intermolecular α-alkylation of aldehydes.

The main reasons for the choice of [Ru(bpy)_3_]^2+^ are: (i) visible light absorption, (ii) chemically reversible electron transfer processes, (iii) sufficiently long-lived T_1_ excited state to be involved in bimolecular reactions and efficient population of the T_1_ state upon visible-light excitation, (iv) photostability of the complex. The photoreactive state is T_1_ that is populated with unitary efficient intersystem crossing from the S_1_ state; T_1_ is a metal-to-ligand charge transfer (MLCT) excited state and metal-centred (MC) states are sufficiently high in energy that they do not contribute to the photoreactivity and possible photodecomposition processes.

The family of investigated metal complexes has been widened to Ir(iii) complexes, which are more powerful photoreductants compared to Ru(ii) polypyridine complexes, and then to earth-abundant metals, like Cu(i), Fe(ii), Co(iii). With earth-abundant metals of the first transition row, the ligand field is decreased for the more contracted nature of 3d orbitals compared to the 4d- and 5d-orbitals and MC excited states are easily accessible; as a consequence, the metal complexes are less photostable and with shorter lifetime of the lowest excited state. A detailed discussion is outside the scope of the present review and interested readers can refer to existing literature.^46–48^

As in the case of the prototypical [Ru(bpy)_3_]^2+^, most of the investigated metal complexes exhibit photoreactivity only from the lowest triplet excited state because of very high efficiency of isc to the lowest-energy excited state, enabled by small S_1_–T_1_ energy gaps and strong spin–orbit coupling associated with heavy metal centres.

Organic photocatalysts have been more recently applied to photoredox catalysis and we can divide them in two families: (i) chromophores with very low efficiency of T_1_ population, or with T_1_ state that is too low in energy to promote the reaction under analysis; (ii) chromophores with good efficiency of T_1_ population, and in which both S_1_ and T_1_ can participate in the photoreaction.

The former class is constituted, for example, by highly fluorescent organic chromophores (coumarin, perylenediimide, BODIPY),^49–51^ in which the S_1_ excited state mainly deactivates by fluorescence decay in the absence of quencher and very inefficient population of T_1_ is observed. These chromophores are employed for the production of value-added chemicals, and less frequently used for the production of solar fuels in homogeneous catalysis because of the need for very high concentration of the quencher. Additionally, if the T_1_ state does not possess a sufficiently high excited-state redox potential for the intended transformation, the photocatalyst, regardless of its isc efficiency, will display only S_1_ reactivity.^52–56^

On the other hand, organic dyes with efficient population of T_1_ state are widely employed both for added-value chemicals^31,57,58^ and artificial photosynthesis.^25,59–62^ This family comprises organic chromophores displaying the lowest in energy transitions as charge-transfer, among which TADF chromophores. When both S_1_ and T_1_ can participate in the photocatalytic cycle (class ii photocatalysts), quenching occurring from S_1_ and T_1_ are two competing pathways: only S_1_ is populated *via* light irradiation, and it can then deactivate either by interaction with the quencher, or *via* isc to populate T_1_, which is the first step that allows T_1_ reactivity.^63^ As mentioned above, electron transfer from T_1_, provided that the process is thermodynamically favourable, is generally more productive than quenching from S_1_, because cage escape from the triplet is generally higher than from the singlet. At the same time, population of T_1_ is bound to the intersystem crossing efficiency, requiring an extra step to allow T_1_ reactivity.

Therefore, depending on the kinetic characteristics of the system, reactivity from T_1_ may be more effective than reactivity from S_1_, or *vice versa*.

## 4. Investigation of the photochemical mechanism

The study of the photochemical mechanism proceeds *via* multiple steps, as summarized below and in Fig. 2.

**Fig. 2:**
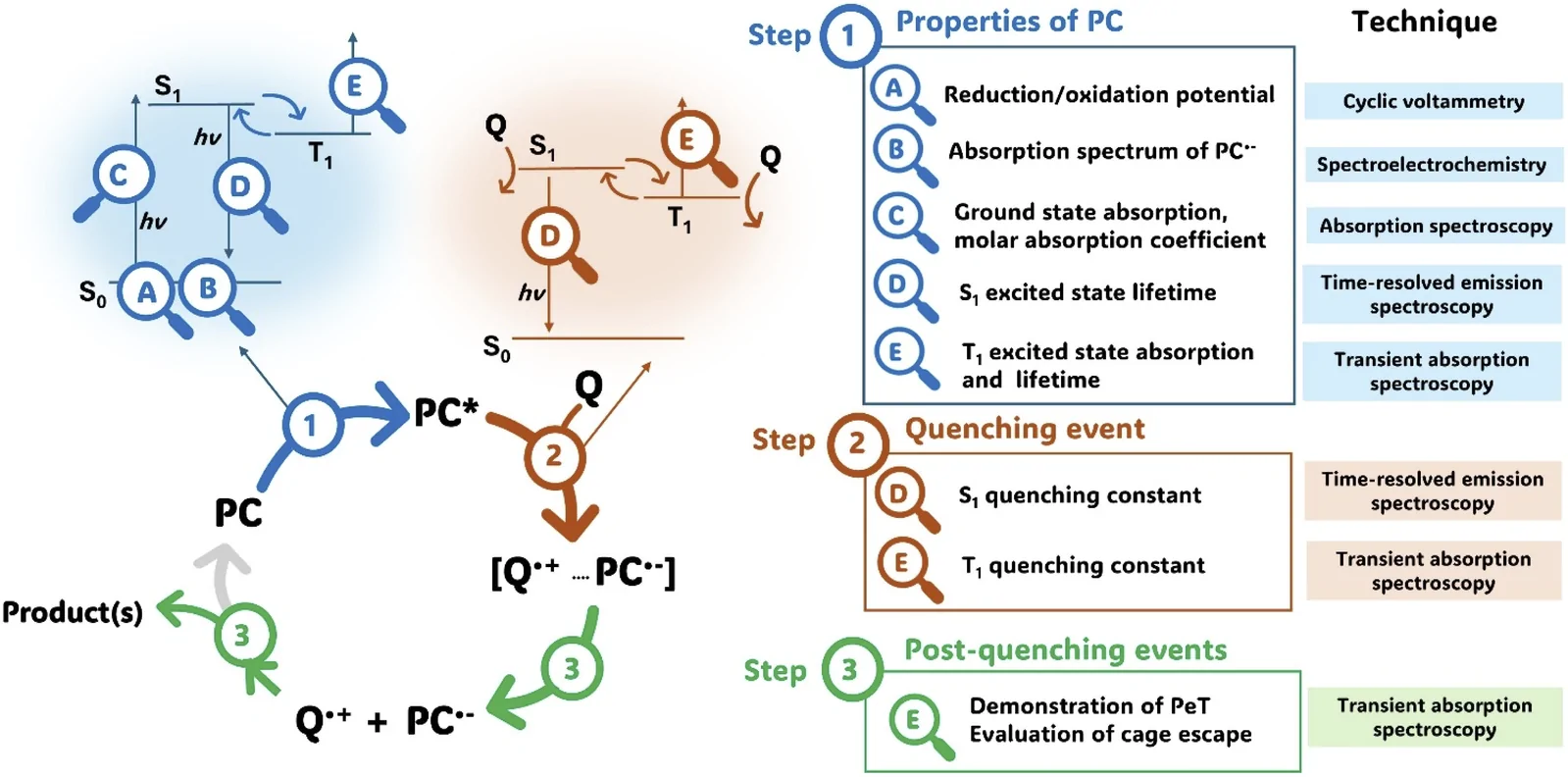
Schematic representation of the steps for a comprehensive investigation of the photocatalytic mechanism. The scheme follows the workflow described in the main text and correlates each photophysical information with the corresponding experimental technique(s). For simplicity, the mechanism is depicted assuming reduction of the photocatalyst (PC) upon quenching; however, the same principle applies to the corresponding oxidative pathway. The reverse intersystem crossing process, explicitly indicated by the arrow from T_1_ to S_1_, is considered operative only for compounds exhibiting TADF. In such cases, process D (fluorescence) can also be exploited to indirectly probe triplet state quenching, making transient spectroscopy (E) not strictly necessary for that step.

(i) Photophysical and electrochemical properties of the photocatalyst under the same solvent and temperature conditions used in the reaction setup (Fig. 2, step 1A–E). An optimal photocatalyst is characterized by large molar absorption coefficient in the employed spectral window of irradiation and high photostability in order to minimize the required concentration of the photocatalyst, long lifetime of the photoreactive states to minimize the concentration of the quencher used in reaction (2) and thus enable reaction (3).

(ii) Evaluation of quenching rate constants and efficiencies of the photocatalyst excited states by each of the reagents used in the reaction setup (Fig. 2, step 2D and E); when possible synergistic effects (*e.g.*, acid–base Lewis effect) are envisaged, evaluation of quenching is performed by mixtures of two or more reagents. The quenching constant *k*_q_ of reaction (2) is evaluated *via* the Stern–Volmer equation:12
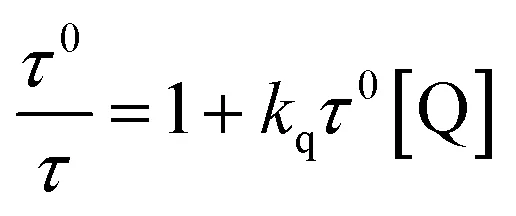
where *τ*^0^, *τ* are the lifetimes of the photoreactive excited state *PC in the absence and in the presence of Q, respectively. The quenching efficiency can be evaluated by eqn (5).

Therefore, to study the quenching efficiency the lifetime of the photocatalyst upon addition of different amounts of quencher must be measured. Most photocatalysts display either fluorescence or phosphorescence. While this is not a prerequisite, it is highly advantageous for the study of photochemical mechanism. Indeed, the concentration of S_1_ and T_1_ excited states is directly proportional to the fluorescence and phosphorescence intensity, respectively. Therefore, lifetime of S_1_ and T_1_ can be easily measured *via* emission intensity decay. The Stern–Volmer eqn (12) can also be expressed in terms of emission intensities rather than lifetimes. This approach can be particularly appealing to research groups without access to transient emission instrumentation and is therefore often used as an alternative to lifetime-based Stern–Volmer analysis.^64,65^ However, obtaining meaningful results from emission intensities requires several stringent conditions to be met, including a linear detector response and negligible inner-filter effects, among others.^6^ If these conditions are not satisfied, the resulting Stern–Volmer analysis may be misleading or invalid. More importantly, for TADF photocatalysts, the complex interplay between singlet and triplet excited states means that emission intensity does not directly report the kinetics of either state. Consequently, lifetime-based Stern–Volmer analysis is more reliable for accurately characterising excited-state quenching in TADF photocatalysts.^27,66^

When the excited state under analysis is non-luminescent (*e.g.*, T_1_ state of most organic chromophores in fluid solution), it is necessary to employ transient absorption spectroscopy. This technique enables the measurement of the electronic absorption spectrum of transient species, such as excited states. The detected signal is proportional to the concentration of the transient species under investigation,^6,67,68^ and therefore can be used to measure the lifetime of the excited state when the latter is not luminescent. However, this technique is experimentally more demanding and less user-friendly than emission spectroscopy, requiring specialized instrumentation and expertise; consequently, it is less widely available. As previously noted, the quenching constant provides information only on the initial bimolecular quenching steps (eqn (8) and (9)) and does not account for subsequent processes such as geminate recombination or cage escape. Therefore, the evaluation of the quenching efficiency alone is not enough to understand the whole catalytic cycle.

(iii) Demonstration of the occurrence of the photoinduced electron transfer step *via* detection of spectroscopic signatures of the radical anion of PC or radical cation of Q in the case of reductive quenching (eqn (2), Fig. 2, step 3E) (the opposite applies for oxidative quenching) *via* transient absorption spectroscopy and comparison of the so-obtained spectrum with the absorption spectra of reduced and oxidized species obtained by spectroelectrochemistry.^69^

(iv) Evaluation of cage escape. As previously discussed, we expect to observe higher cage escape efficiency from the quenching of T_1_ than S_1_ excited state and direct evaluation of this parameter can be obtained by transient absorption spectroscopy, as described in ref. 67. Transient absorption spectroscopy can also be used to study the fate of other radical species involved in the reaction mechanism, such as A˙^−^, provided that the lifetime of these radicals is compatible with the time-resolution of the system and that its rate of formation is faster than its rate of consumption (Fig. 2, step 3E).

(v) Determination of the overall photoreaction quantum yield (*Φ*_P_) and comparison with the quenching quantum yield: when the photoreaction quantum yield is higher than the quenching quantum yield, a chain reaction is in place. It is worth noting that the photoreaction quantum yield is expected to change during the reaction because the efficiency of the photoinduced electron transfer process (eqn (2)) depends on the quencher concentration and, when this is a sacrificial reagent, it is consumed during the reaction.

(vi) Determination of the photostability of the photocatalyst in the experimental condition used, also by recovery of the photocatalyst at the end of the reaction. This step might allow to optimize the reaction conditions and diminish the photocatalyst concentration.

Additionally, the photon flux might play an important role in the reaction because the higher the photon flux the higher the concentration of radical species, *e.g.* A˙^−^ in reaction (3), formed per unit time. Indeed, the photon flux modulates the steady-state concentration of radical intermediates, thereby affecting the balance between productive pathways and deactivation processes such as radical recombination or back electron transfer.^70,71^

## 5. Influence of S_1_ and T_1_ reactivity over the overall photoreaction quantum yield and relevant case studies

In this section, we will examine some literature examples that show how a deep understanding of the system, particularly in terms of differences in S_1_ and T_1_ reactivity, can favour a smarter design of a photoreaction. The selection of examples has been made based on their educational value, with particular attention devoted to TADF photocatalysts, which are widely employed and characterized by more complex kinetic behaviour.

### 5.1 Dependence of *Φ*_P_ on S_1_ and T_1_ quenching efficiency

In 1992, Ito *et al.* studied the photoreactivity of S_1_ and T_1_ excited states of C_60_ with three amines. Through transient absorption spectroscopy, they measured the amount of C_60_˙^−^ formed by photoinduced electron transfer at varying concentrations of the amine.^72^ They showed that C_60_˙^−^ formation is disfavoured at high amine concentrations. The authors rationalized this observation by proposing that radical pairs generated from the triplet state of C_60_ undergo cage escape more efficiently than those generated from its singlet state, although no quantitative cage-escape efficiency was reported.

A similar trend was subsequently observed by Ishitani *et al.* in CO_2_ photoreduction using the TADF photocatalysts (*e.g.* 4DPAIPN, Fig. 3) and a non-TADF N-BAP^+^ derivative (Fig. 3).^25,73^ All these photocatalysts can react through both S_1_ and T_1_. Despite their different photophysical properties, the photocatalysts showed the same counterintuitive behaviour: increasing the initial quencher concentration ([Q]_0_) resulted in a decrease in the overall photoreaction quantum yield. This was attributed to the different cage-escape efficiencies of the radical pairs formed from the two excited states, with *η*_ceT_/*η*_ceS_ > 8 in ref. 25 and *η*_ceT_/*η*_ceS_ > 18 in ref. 73. Thus, at lower quencher concentrations, the more productive triplet quenching pathway is favoured, whereas increasing the quencher concentration progressively enhances the less efficient singlet pathway. In this case, the cage-escape efficiencies were not measured directly, but estimated from fitting of the concentration-dependent reaction yields. This approach provides a valuable connection between the early photophysical steps and the overall catalytic performance, although it does not allow the different contributions of primary geminate in-cage recombination and bulk (out-of-cage) recombination to be experimentally separated.

**Fig. 3:**
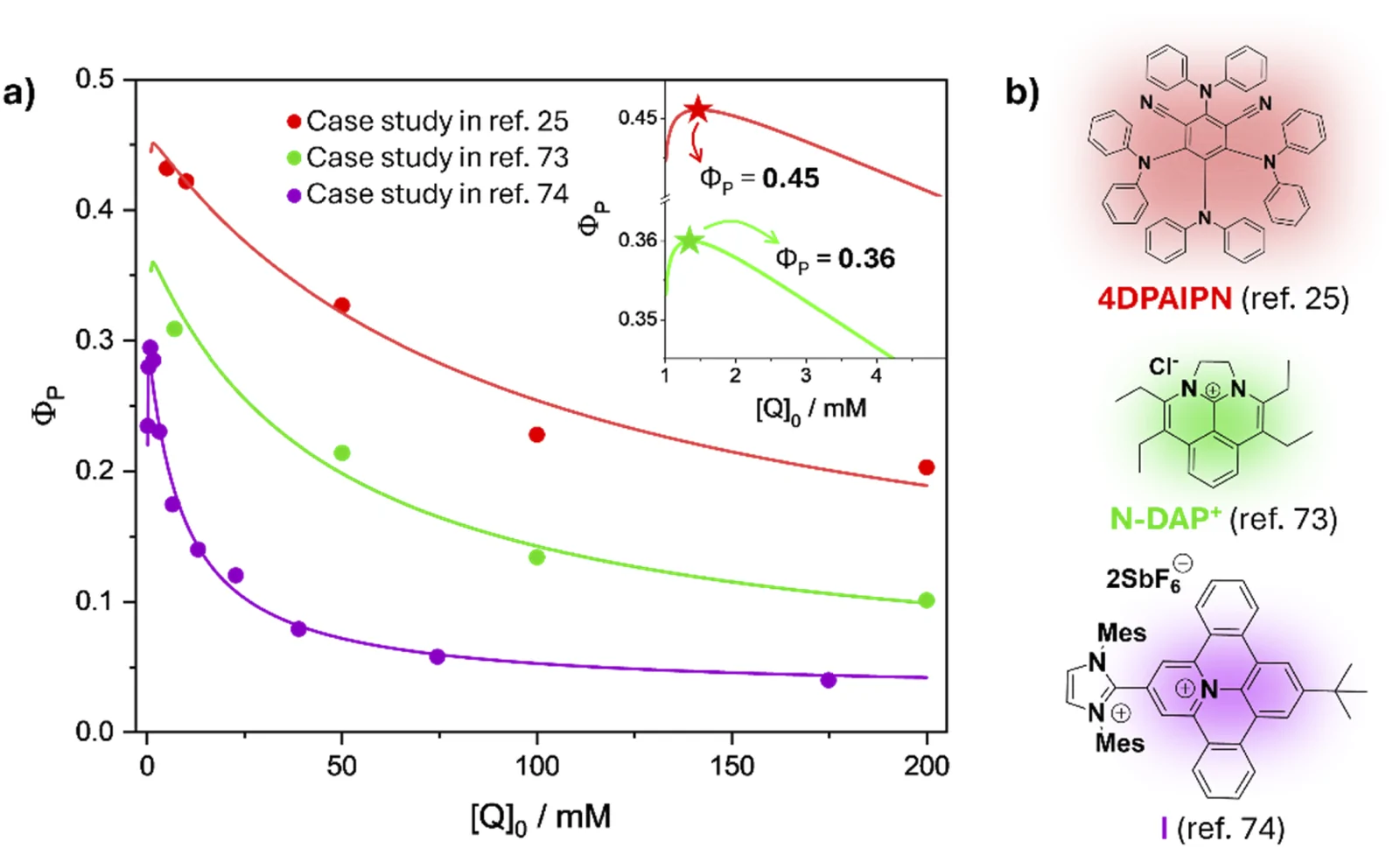
(a) Comparison between the experimental data reported in ref. 25, 73 and 74 (scatter plots) and the kinetic model reported in ref. 27 (line plots). The inset shows an enlarged view of red and green curve in the main graph, highlighting the predicted maximum of *Φ*_P_ with a star. (b) Chemical formulas of the photocatalysts under analysis in ref. 25, 73 and 74.

More recently, Kerzig *et al.*^74^ provided a direct experimental quantification of this difference in cage-escape efficiency. They observed a similar concentration dependence for three photocatalysts undergoing photoinduced electron transfer from both S_1_ and T_1_, using different quenchers (4-*tert*-butylcyclohexanol, naphthalene, dimethylaniline, ascorbate). Upon increasing quencher concentration, the quantum yield of free radical formation decreased, consistently with the trend reported by Ito and Ishitani. Importantly, the cage-escape efficiencies of singlet- and triplet-derived radical pairs were directly determined by transient absorption spectroscopy, monitoring the free radicals formed immediately after cage escape (process (10)) and prior any out-of cage back electron transfer (process (4)). This resulted in *η*_ceT_/*η*_ceS_ values ranging from 12 to 125. These results provide a quantitative basis for the observed concentration dependence: when electron transfer from T_1_ is significantly more productive than quenching from S_1_ (*η*_ceT_ ≫ *η*_ceS_), increasing the quencher concentration can favour the less efficient S_1_ pathway and consequently decrease the yield of freely diffusing radicals.

The relevance of this picture to preparative photochemistry was further illustrated by the observation of the “less is more” effect in the pinacol coupling of acetophenone using SO_2_TDS^2−^ (Fig. 3) as photocatalyst and ascorbate as reductive quencher under 390 nm LED irradiation. The reaction yield was ∼50% higher at a lower quencher concentration (19.2 mM) than with a larger excess of the quencher (96.9 mM), in good agreement with the relative SO_2_TDS˙^3−^ yields determined spectroscopically.

Together, all these studies establish a consistent picture in which the concentration of the quencher can affect the balance between singlet- and triplet-derived pathways and, ultimately, the photochemical efficiency and highlight the predictive power of photophysical studies to anticipate reaction performances.

In addition to these experimental evidence, our group recently developed a kinetic model^27^ that predicts the same behavior theoretically and can, in principle, be applied to any system for which all kinetic rate constants are known. The model (line plots in Fig. 3) shows excellent agreement with all the experimental data analysed, supporting its applicability to real systems.

The kinetic model demonstrates a key condition to observe the counterintuitive dependence of *Φ*_P_ on [Q]_0_:13
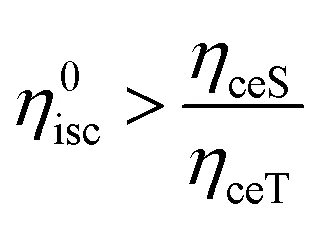
where *η*^0^_isc_ is the efficiency of isc of the photocatalyst without the quencher. If the photocatalyst-quencher couple respects condition eqn (13) – as in all case studies analysed here^25,72–74^ – T_1_ reactivity should be preferred over S_1_ reactivity.^27^ If this is the case, an optimal quencher concentration can be found such that S_1_ is only slightly quenched, while the majority of S_1_ evolves to T_1_ state, which becomes the predominantly active catalytic state.

Back electron transfer (reaction (4)) was not considered in the present kinetic model, in agreement with literature by Ishitani *et al.*,^25^ who observed that the oxidized quencher BIH˙^+^ undergoes a very rapid proton loss that outcompetes back electron transfer. This trend with a maximum in the photoreaction quantum yield, as displayed in Fig. 3, will be maintained even if back electron transfer (reaction (4)) is not negligible, the only difference will be that the photoreaction quantum yield will be reduced by a percentage equal to the efficiency of the back electron transfer process.

It is worth discussing an example in which the photoreaction quantum yield was not dependent on the spin state of the photocatalyst and condition eqn (13) was not in place, although not directly measured. In atom transfer radical polymerisation using phenoxazine photocatalysts and diethyl 2-bromo-2-methylmalonate as the initiator (quencher Q),^75^ the same photoreaction quantum yield was measured for phenoxazine photocatalysts reacting either from the S_1_ or the T_1_ state.‡‡It should be noted, however, that this comparison involved different photocatalysts reacting from the two excited states, rather than the S_1_ and T_1_ states of the same photocatalyst. This behaviour is likely related to the concerted dissociative electron-transfer mechanism reported by Savéant,^76,77^ characteristic of alkyl halides, in contrast to the stepwise mechanism commonly observed for aryl halides. Because electron transfer is accompanied by C–Br bond cleavage, the primary products formed within the solvent cage are PC˙^+^, a carbon-centered radical, and Br^−^. This suppresses geminate charge recombination (reaction (11)) from both S_1_ and T_1_ state quenching, allowing exceptionally high values of cage escape from both states.

The kinetic model predicts that the photoreaction quantum yield exhibits an S-shaped dependence on the ratio of the quenching constants of T_1_ and S_1_ (Fig. 4). When S_1_ is quenched several orders of magnitude faster than T_1_, the reaction is dominated by S_1_ reactivity even under optimized conditions, with optimal *Φ*_P_ approaching *η*_ceS_. This regime occurs when T_1_ lacks sufficient redox power to drive fast electron transfer processes (family (i) in Section 3); as a result, there is essentially no competition between the two states, and maximizing S_1_ quenching with high concentration of quencher is most beneficial. As the quenching rate constant of T_1_ increases, the overall reaction efficiency under optimized conditions improves. In this intermediate regime, it becomes progressively easier to favour T_1_ over S_1_ reactivity by lowering the optimal quencher concentration [Q]. In the limiting case where T_1_ is quenched more efficiently than S_1_, *Φ*_P_ approaches *η*_isc_·*η*_ceT_, and the performance becomes governed primarily by the intersystem crossing and T_1_ cage escape, which act as bottlenecks. Thus, increasing *k*_qT_ drives a transition from S_1_-dominated to T_1_-dominated reactivity. The position of this transition region (highlighted in purple in Fig. 4) depends on the difference between the intrinsic (unquenched) time constants of S_1_ and T_1_. The larger this difference, the easier it is to achieve high *Φ*_P_ even when T_1_ is only weakly quenched, since its higher population can compensate for a lower *k*_qT_.

**Fig. 4:**
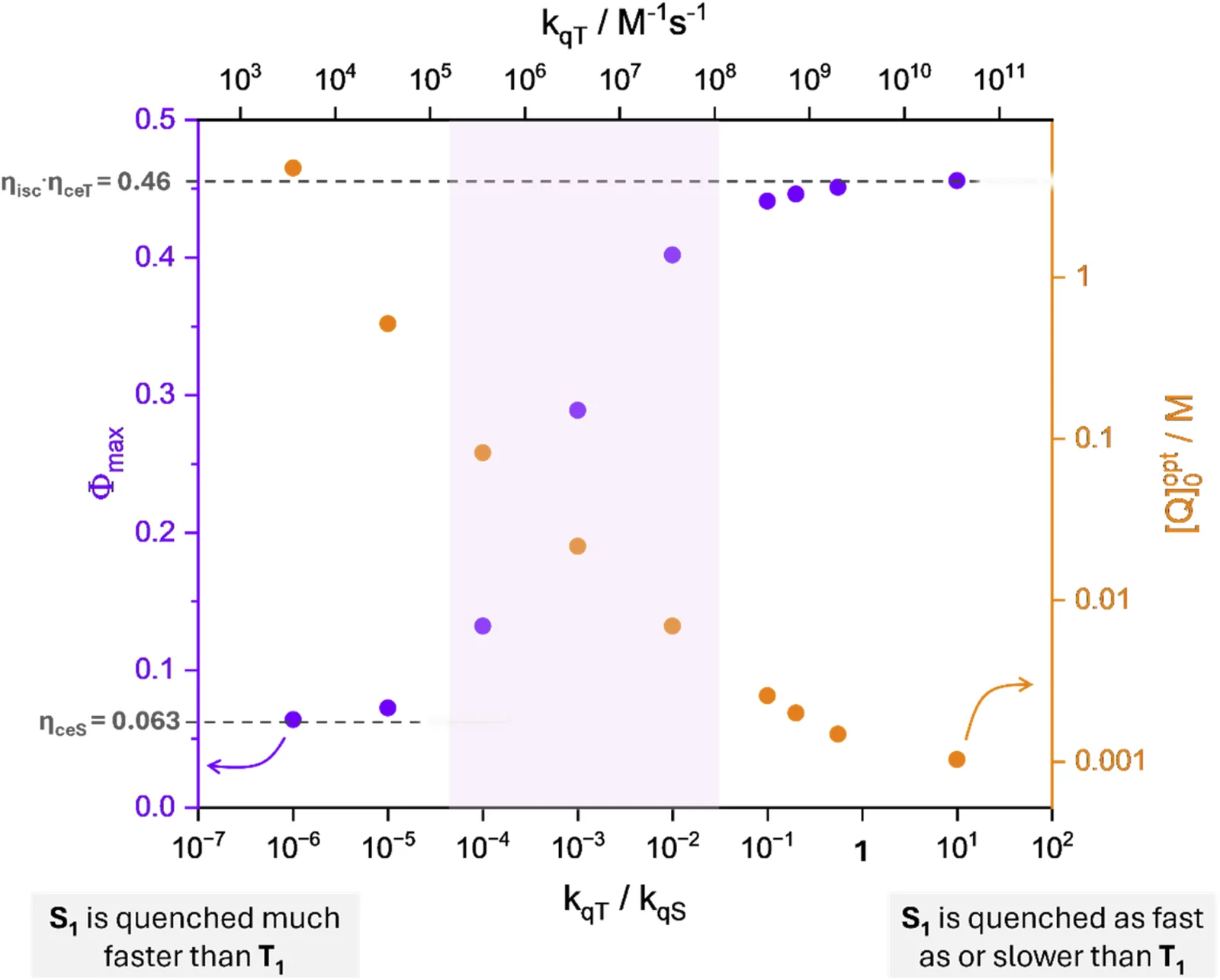
Variation of *Φ*_P_ (purple plot) calculated according to ref. 27 by changing the value of *k*_qT_. All other kinetic constants are taken as those reported for 4DPAIPN in ref. 25. *Φ*_P_ is calculated for the optimal concentration of quencher [Q]^opt^_0_, which is also reported in the graph (orange scatter plot).

These considerations are quite counterintuitive, with many previous reports stating that a high quencher concentration should in general be preferred. For example, this condition is suggested in the work of Swierk *et al.*,^78^ where the difficulty of intercepting short-lived excited states is explicitly addressed. The study advocates optimization of quencher concentration and identifies approximately 90% fluorescence quenching as a practical compromise, implying that, because nanosecond singlet lifetimes make bimolecular quenching kinetically challenging, quencher loadings often need to be raised well above the concentrations commonly used in literature to achieve the optimal *Φ*_P_. However, in a literature report based on acridinium photocatalysts,^79^ increasing quencher concentration from 80 to 160 mM led to a significant decrease in reaction yield, from 0.43 to 0.33. This behaviour is not commented in the reference text but may reflect an involvement of a triplet-mediated reaction pathway. In fact, other acridinium derivatives have shown non-negligible population of T_1_ state.^56,80^ Similar effects have been observed for the isomerization of *cis*-stilbene sensitized by pyrylium salts, initiated by a photoinduced electron transfer from stilbene to the excited sensitizer, generating a stilbene radical cation as the key reactive intermediate.^81,82^ While both the excited singlet and triplet states of the photocatalyst are quenched by stilbene at diffusion-controlled rates, productive *Z*-to-*E* isomerization appeared to occur only through the triplet pathway, as evident from the fact that the isomerization quantum yield increased as the concentration of stilbene decreases.

For these reasons, luminescence quenching that has been proposed as a rapid, “mechanism-based screening” method^83^ to identify promising substrate classes by testing combinations of quenchers and photocatalysts may be potentially deceptive. This approach can be useful when comparing photocatalysts belonging to the same class, where there is only one photocatalytically active state – either S_1_ or T_1_. When a possible interplay within states of different multiplicity cannot be excluded, a more comprehensive approach should be preferred instead of relying on luminescence quenching efficiency as proxy for quantum efficiency. In this case, proposing a single quenching efficiency cutoff across different photocatalysts oversimplifies the treatment: each system is characterized by its own kinetic constants, the same percentage of luminescence suppression does not necessarily correspond to the same effect on triplet population and therefore on productive electron transfer.

### 5.2 Change of *Φ*_P_ during reaction progress

Another important aspect to consider is that the photoreaction quantum yield may vary significantly over the course of a reaction (Fig. 5). Traditionally, attention has focused almost exclusively on decreases in quantum yield,^9,70,84^ which are commonly interpreted as evidence of photocatalyst degradation. For this reason, quantum yields are generally calculated in the early stages of photoreactions, to avoid artifacts related to side reactions.^9,25,59,73,84,85^ On the other hand, in an ideal reaction batch – with no side-reactions involved – the quantum yield can change as a result of a significant decrease in the concentration of the quencher, which affects the quenching efficiency of S_1_ and T_1_. The effect of this decrease over the photoreaction quantum yield is not always negative: the discriminant is again condition eqn (13). If the latter is satisfied, and if the initial quencher concentration is higher than the optimal condition, the photoreaction quantum yield will increase over time (see, as an example, the calculated *Φ*_P_ for [Q]_0_ > 1.4 mM in Fig. 5). On the other hand, if the initial concentration is set at the optimal value or less, the photoreaction quantum yield will generally decrease (calculated *Φ*_P_ for [Q]_0_ ≤ 1.4 mM in Fig. 5). Either way, a correct evaluation of the photoreaction quantum yield should consider these factors, and the experimental conditions of the photoreaction should be considered when reporting the value of *Φ*_P_. This analysis can (i) improve the fundamental understanding of the mechanism governing photoreactivity, and (ii) suggest easy optimization methods: if *Φ*_P_ increases with time, a decrease in [Q]_0_ will likely improve the efficiency of the system; on the other hand, if a decreasing trend is observed over time, the optimal [Q]_0_ is likely higher than the value employed. Flow setup which keeps the quencher concentration at the optimal value can also be engineered.

**Fig. 5:**
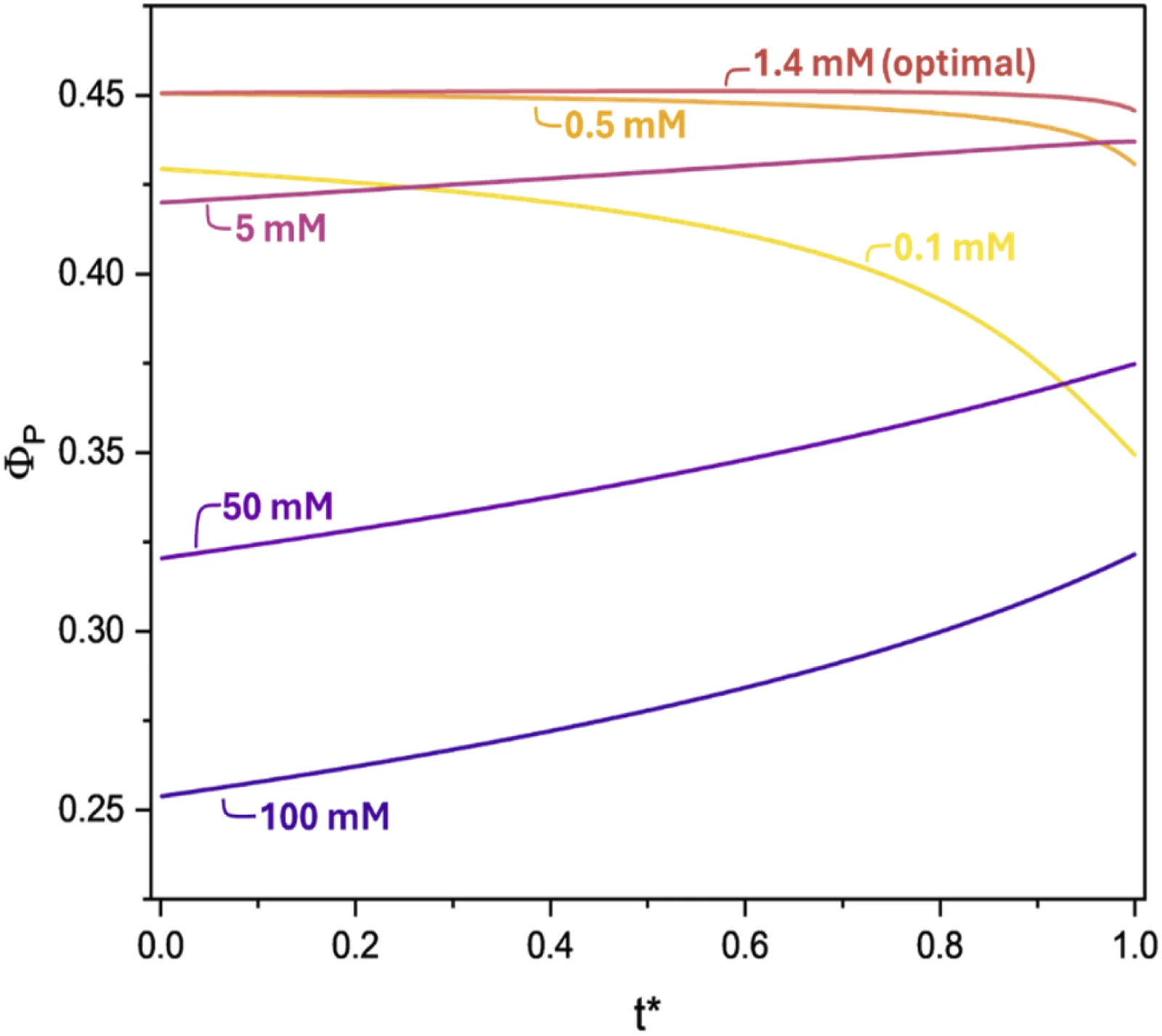
Evolution of the photoreaction quantum yield (*Φ*_P_) along normalised time t* as calculated with the kinetic simulator reported in ref. 60 (t* = Δ*t*/Δ*t*_fin_, with Δ*t*_fin_ defined as the time corresponding to [Q]_*t*_ = 0.01[Q]_0_). We used the same kinetic constants reported for 4DPAIPN in ref. 25. For this system, the calculated optimal concentration of quencher is [Q]_0_ = 1.4 mM.

### 5.3 The role of TADF in photocatalysis

Thermally activated delayed fluorescence (TADF) photocatalysts have become increasingly prominent in organic photoredox catalysis, owing to their ability to efficiently populate long-lived triplet excited states while retaining the possibility of thermally activated repopulation of the singlet state. However, despite their widespread use, the question of whether TADF itself provides an intrinsic advantage for photocatalysis remains open.^86^ In other words, is the presence of efficient reverse intersystem crossing beneficial to photocatalytic reactivity, or is TADF merely a consequence of the combination of photophysical properties that make these compounds effective photocatalysts? This question becomes particularly interesting considering the recent emergence of metal-based TADF photocatalysts.^87–90^ In contrast to conventional metal-based photocatalysts, for which the lowest triplet excited state is generally considered the only relevant reactive state, TADF metal complexes introduce an intrinsic competition between S_1_ and T_1_ reactivity.

A first important difference between the two classes concerns the rate of isc. In TADF organic photocatalysts, intersystem crossing can be highly efficient (*η*_isc_ = 0.5–0.9 with *k*_isc_ ∼ 10^8^ s^−1^),^25,31,40,59,74^ but is generally slower than in many metal-containing systems, where spin–orbit coupling can promote extremely rapid S_1_ → T_1_ conversion (*k*_isc_ > 10^9^ s^−1^ for many metal complexes employed in photocatalysis). This difference is particularly relevant under photocatalytic conditions where isc competes directly with quenching of S_1_. The relevant parameter is therefore the kinetic competition between *k*_isc_ and the pseudo-first-order quenching rate *k*_qS_[Q]. Even a photocatalyst displaying essentially quantitative isc in the absence of quencher can undergo substantial S_1_ quenching at the relatively high quencher concentrations commonly employed in photocatalysis. For example, if *k*_isc_ = 10^8^ s^−1^, *k*_qS_ = 10^9^ M^−1^ s^−1^, and [Q] = 0.1 M, the corresponding S_1_ quenching rate is 10^8^ s^−1^, making isc and S_1_ quenching equally competitive: half of the initially populated S_1_ states undergoes S_1_ quenching, hindering T_1_ reactivity. By contrast, metal-based TADF systems displaying *k*_isc_ values in the 10^9^–10^11^ s^−1^ range can efficiently populate T_1_ state even at high quencher concentrations. This aspect is highlighted in Fig. 6, where we compared the calculated performances of two representative systems: 4DPAIPN^25,31^ and Cu^MAC^_BCz_, a reported carbene–copper–amide complex displaying TADF, recently used as photocatalyst in hydrogen production.^91^ We calculated *Φ*_P_ over a large [Q] range according to the kinetic constants reported in the relevant references (ref. 25 for 4DPAIPN and ref. 91 for Cu^MAC^_BCz_). The black curves in Fig. 6a and b show that the very rapid isc characteristic of Cu^MAC^_BCz_ and similar complexes can be extremely advantageous when large *k*_qS_ values and/or high quencher concentrations are employed (black lines in Fig. 6, high [Q] region), whereas organic TADF photocatalysts such as 4DPAIPN may perform equally well only when lower quencher concentrations and less efficient S_1_ quenching are in place (black lines in Fig. 6, low [Q] region).

**Fig. 6:**
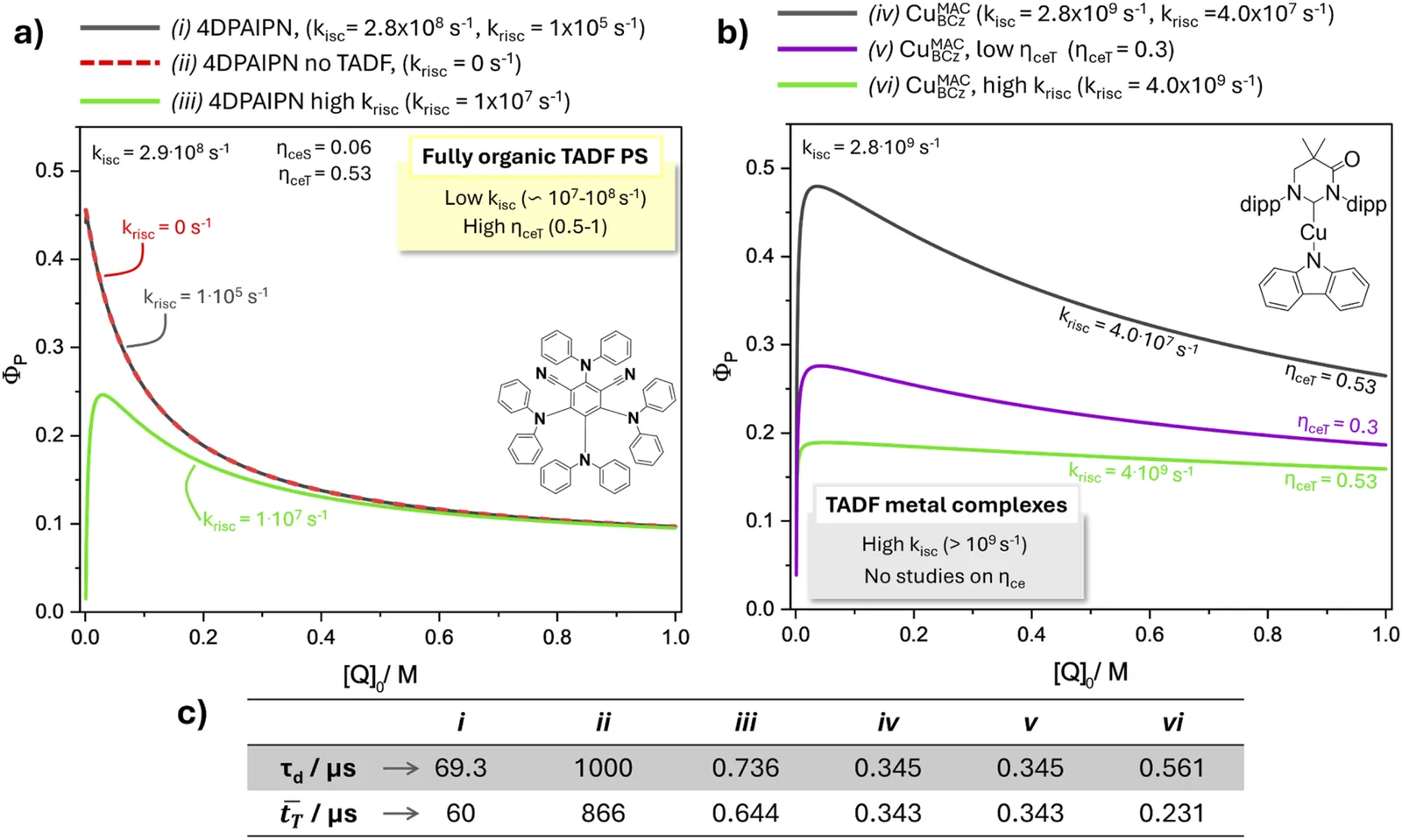
Comparison of *Φ*_P_ calculated according to ref. 27 over different [Q]_0_ for six different sets of kinetic constants. (a) Kinetic curves calculated using the kinetic constants of 4DPAIPN and the quenching constants and cage escape efficiencies as reported in ref. 25, without modification (black curve), or by setting *k*_risc_ = 0 s^−1^ (red dashed curve) or 1 × 10^7^ s^−1^ (green curve); (b) kinetic curves calculated using the kinetic constants of Cu^MAC^_BCz_ as reported in ref. 91 and the quenching constants and cage escape efficiencies used in the left panel (black curve) or by setting *η*_ceT_ = 0.3 (purple curve) or *k*_risc_ = 1 × 10^7^ s^−1^ (green curve); (c) calculated values of triplet lifetime (*τ*_d_) and triplet residence time (*t̄*_T_) for cases i–vi. 4DPAIPN and Cu^MAC^_BCz_ structures are reported in panel (a) and (b), respectively.

As discussed above, the cage escape efficiency is another important parameter to consider. Notably, studies on TADF metal complexes rarely report cage escape efficiencies. As illustrated in Fig. 6b, curve *v*, also in metal complexes a decrease of the cage escape efficiency results in a pronounced decrease in the overall photoreaction quantum yield in the whole [Q] range analysed. These results highlight the need for studies of metal-based TADF photocatalysts to systematically include state-specific cage escape efficiencies quantification alongside other key photophysical parameters.

The role of risc for catalysis is more subtle. If T_1_ is the more productive state for photocatalysis, risc does not intrinsically promote the photoreaction. Rather, it removes population from the state in which productive photochemistry preferentially occurs. This can be expressed by considering the residence time in T_1_ (*t̄*_T_, the average time the molecule resides in T_1_ excited state), which decreases upon increasing *k*_risc_:14
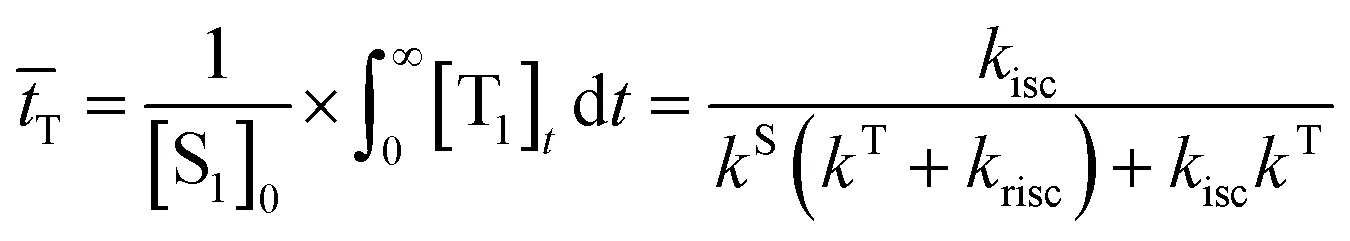
where *k*^S^ = *k*_fl_ + *k*_ic_ and *k*^T^ = *k*_ph_ + *k*_isc′_ (see Fig. 1).

Importantly, this should not be confused with the overall excited-state lifetime: risc can redistribute population between S_1_ and T_1_ and thereby extend the total lifetime of the excited-state manifold, while simultaneously decreasing the time spent in T_1_. From the perspective of photocatalysis, the relevant quantity is not simply how long the photocatalyst remains electronically excited, but how long it resides in the state that undergoes productive quenching and subsequent photochemistry, namely T_1_.

This distinction is illustrated by the kinetic simulations depicted in Fig. 6. Comparison of the reference 4DPAIPN system^25^ with a hypothetical analogue in which *k*_risc_ = 0 s^−1^ shows that suppressing risc increases the T_1_ residence time (Fig. 6c) and consequently slightly enhances the photoreaction quantum yield, particularly at low quencher concentrations (Fig. 6a, cases i and ii). Conversely, a substantial increase of *k*_risc_ decreases the T_1_ residence time and leads to lower photoreaction quantum yields (case iii in Fig. 6a). The same behaviour is observed for the system mimicking Cu^MAC^_BCz_: in this case, even if imposing very high values of *k*_risc_ increases T_1_ lifetime as a consequence of the continuous cycling between S_1_ and T_1_, the residence time on T_1_ decreases (case vi in Fig. 6c), and therefore photocatalytic performances are lower than those recorded for the reference system (cases iv and vi in Fig. 6b).

Overall, these considerations indicate that TADF is not intrinsically beneficial to photocatalysis. A natural question at this point is why TADF photocatalysts have become so successful in recent years. While this is a fair and important point, the explanation does not lie primarily in their TADF character itself. Rather, their effectiveness in photocatalysis comes from the sum of concomitantly advantageous features: TADF photocatalysts offer efficient T_1_ population with relatively long lifetimes, and they ensure small energy losses when T_1_ is involved rather S_1_, broadening the scope of substrates available for the catalytic cycle. In addition to that, common TADF photocatalysts based on cyanoarene core are easy to synthesize and their scaffold is easily tunable in terms of optoelectronic properties, which makes them a highly versatile option.^31,57,92^ The TADF phenomenon is a natural result of the sum of such photophysical properties, although it has little effect on the photocatalytic performances. The same reasoning applies to metal-based TADF photocatalysts: their exceptionally rapid isc can be advantageous because it protects S_1_ from competitive quenching under conditions of high *k*_qS_[Q], but risc itself does not provide an intrinsic photocatalytic benefit. Rather, the balance between isc, S_1_/T_1_ quenching, T_1_ residence time, and radical-pair cage escape ultimately determines their high photocatalytic performances.

However, TADF photocatalysts show an advantageous aspect with respect to traditional photocatalysts in terms of mechanistic analysis: risc – exclusive of photocatalysts displaying TADF – allows to probe all the processes occurring at T_1_ through delayed fluorescence. In fact, it has previously been shown that the quenching constant measured on the prompt and delayed components of the fluorescence decay mirror the quenching constants of S_1_ and T_1_, respectively.^27,33^ Therefore, mechanistic studies on TADF-based photocatalytic are more accessible, since the study of triplet reactivity does not rely on transient absorption spectroscopy, requiring higher degree of expertise and less available equipment.

## 6. Conclusions and future perspectives

The optimization of photocatalytic efficiency relies on a synergy between the rational photocatalyst design and the rigorous control of experimental conditions. While early developments in the field primarily utilized metal complexes characterized by nearly unitary intersystem crossing and predictable T_1_ reactivity, the shift toward organic photocatalysts, and particularly TADF systems, has introduced a more complex kinetic interplay between S_1_ and T_1_ channels. This complexity necessitates moving beyond pure trial-and-error approaches in the top-down optimization of catalytic cycles, as well as beyond simplified screening methods entirely based on the efficiency of luminescence quenching, which can be misleading when multiple reactive states compete. Instead, detailed mechanistic investigations are essential. While emission decay measurements are valuable for studying luminescent states, techniques such as transient absorption spectroscopy are crucial for determining the lifetimes of non-luminescent triplet states, quantifying key parameters such as cage-escape efficiency, which often governs the overall quantum yield, and tracking the fate of the generated radicals.

Achieving these goals requires close collaboration between photochemistry and organic chemistry groups to ensure that kinetic models are effectively integrated into synthetic applications. Finally, greater awareness is required, together with a careful comparison of the different operating conditions under which photochemical measurements and reaction development are conducted, in order to bridge the gap between idealized mechanistic studies and practical synthetic implementations.

## Author contributions

All authors conceived and wrote the whole manuscript. C. B. and F. F. designed and produced the figures, partially elaborating data reported by cited previous reports. All authors contributed to the writing of the paper.

## Conflicts of interest

There are no conflicts to declare.

## Data Availability

No primary research results, software or code have been included and no new data were generated or analysed as part of this review.
